# Surveillance of Diarrheagenic *Escherichia coli* Strains Isolated from Diarrhea Cases from Children, Adults and Elderly at Northwest of Mexico

**DOI:** 10.3389/fmicb.2016.01924

**Published:** 2016-11-30

**Authors:** Adrian Canizalez-Roman, Héctor M. Flores-Villaseñor, Edgar Gonzalez-Nuñez, Jorge Velazquez-Roman, Jorge E. Vidal, Secundino Muro-Amador, Gerardo Alapizco-Castro, J. Alberto Díaz-Quiñonez, Nidia León-Sicairos

**Affiliations:** ^1^CIASaP, School of Medicine, Autonomous University of SinaloaCuliacan, Mexico; ^2^The Women’s Hospital, Secretariat of HealthCuliacan, Mexico; ^3^The Sinaloa State Public Health Laboratory, Secretariat of HealthCuliacan, Mexico; ^4^Hubert Department of Global Health, Rollins School of Public Health, Emory University, AtlantaGA, USA; ^5^Coordinación de Enseñanza e InvestigaciónCuliacan, Mexico; ^6^Instituto de Diagnóstico y Referencia EpidemiológicosMexico City, Mexico; ^7^Facultad de Medicina, Universidad Nacional Autónoma de MéxicoMexico City, Mexico; ^8^Pediatric Hospital of SinaloaCuliacan, Mexico

**Keywords:** diarrheagenic *E. coli*, biosurveillance, antibiotic resistance, diarrhea, pathotypes

## Abstract

Diarrheagenic *Escherichia coli* (DEC) strains are a main cause of gastrointestinal disease in developing countries. In this study we report the epidemiologic surveillance in a 4-year period (January 2011 to December 2014) of DEC strains causing acute diarrhea throughout the Sinaloa State, Mexico. DEC strains were isolated from outpatients of all ages with acute diarrhea (*N* = 1,037). Specific DEC pathotypes were identified by PCR-amplification of genes encoding virulence factors. The adhesion phenotype and antibiotic resistance were also investigated. DEC strains were detected in 23.3% (242/1037) of cases. The most frequently DEC strain isolated was EAEC [(12.2%), 126/242] followed by EPEC [(5.1%), 53/242], ETEC [(4.3%), 43/242] DAEC [(1.4%), 15/242], STEC [(0.3%), 3/242], and EIEC [(0.2%), 2/242]. EHEC strains were not detected. Overall DEC strains were more prevalent in children ≤2 years of age with EPEC strains the most common of DEC pathotypes. While ∼65% of EAEC strains were classified as typical variant based on the aggregative adherence to *in vitro* cultures of HEp-2 cells, a high proportion of EPEC strains was classified as atypical strains. EAEC, EPEC, ETEC, and DAEC strains were distributed in the north, central and south regions of Sinaloa state. Among all DEC strains, >90% were resistant to at least one commonly prescribed antibiotic. Strains were commonly resistant to first-line antibiotics such as tetracycline, ampicillin, and sulfamethoxazole-trimethoprim. Furthermore, more than 80% of DEC isolates were multi-drug resistant and EPEC and DAEC were the categories with major proportion of this feature. In conclusion, in nearly one out of four cases of acute diarrhea in Northwestern Mexico a multi-drug resistant DEC strain was isolated, in these cases EAEC was the most prevalent (52%) pathotype.

## Introduction

Diarrhea remains an important cause of morbidity and mortality globally, particularly among infants and young children. It was estimated that in 2010 there were more than 1.7 billion episodes of diarrhea worldwide from which 700,000 led to death in children under 5 years of age (Liu et al., 2012; Walker et al., 2013). The different pathotypes of diarrheagenic *Escherichia coli* (DEC) are a main cause of diarrhea particularly in developing countries (Nataro and Kaper, 1998). DEC strains have been classified into six groups based on their specific virulence factors and phenotypic traits and include Enteroaggregative *E. coli* (EAEC), Enteropathogenic *E. coli* (EPEC), Enterotoxigenic *E. coli* (ETEC), Enteroinvasive *E. coli* (EIEC), Diffusely adherent *E. coli* (DAEC) and Enterohemorrhagic *E. coli* (EHEC), and shiga-like toxin (STEC) (Kaper et al., 2004).

Diarrheagenic *Escherichia coli* strains have also been involved in outbreaks of gastroenteritis and systemic disease. For example, a STEC strain serotype O104:H4 was responsible in 2011 for the largest epidemic outbreaks in Germany. This multidrug-resistant STEC caused over 3800 cases of diarrhea without hemolytic uremic syndrome (HUS) and over 830 cases with HUS, leading to 54 deaths. Furthermore, STEC O104:H4 infection was observed in 12 other European countries and in North America and Canada (Frank et al., 2011). Although, DEC pathotypes are a main source of diarrhea and therefore of public health relevance, strains are not routinely sought as enteric pathogens in clinical laboratories worldwide. Therefore, prevalence of diarrhea caused by DEC strains are generally unknown, particularly in areas where DEC strains are believed endemic.

In Mexico, the presence of DEC has been evaluated in hospitalized patients (Estrada-Garcia et al., 2005), in children less than 2 years of age (Estrada-Garcia et al., 2009) and some studies in U.S. students traveling to Mexico who were infected with DEC strains (Huang et al., 2003, 2008; Paredes-Paredes et al., 2011). However, there are limited reports investigating the role of DEC pathotypes on acute diarrhea for all age groups (including children, adults and the elderly).

In addition, the treatment for *Enterobacteriaceae* family including *E. coli* has been increasingly complicated by the emergence of resistant strains to most first-line antimicrobial agents in the last decades (Wellington et al., 2013). Here we report on an epidemiologic surveillance study of DEC in patients with acute diarrhea in the northwest state of Sinaloa, Mexico, using a sequential pathogen-specific multiplex PCR, their adherence pattern and antimicrobial resistance profile.

## Materials and Methods

### Bacterial Strains

Diarrheagenic *Escherichia coli* reference strains utilized in this study belong to our laboratory collection (Canizalez-Roman et al., 2013). Target genes and the identified pathotype are the following: enteropathogenic *E. coli* (EPEC E2348/69; *eae*^+^ and *bfp*A^+^), Enterotoxigenic *E. coli* (ETEC; *lt*^+^, *st*^+^), Enteroinvasive *E. coli* (EIEC; *ipa*H^+^, *vir*F^+^), Enterohemorrhagic *E. coli* (EHEC O157:H7 EDL933; *eae*^+^, *hly*A^+^, *stx*1^+^, *stx*2^+^), diffusely adherent *E. coli* (DAEC; *daa*E^+^), enteroaggregative *E. coli* (EAEC O42; *agg*R^+^, *aap*^+^, pCVD432^+^, and *aaf*II^+^). *E. coli* DH5α (Invitrogen), *E. coli* ATCC 25922 and *E. coli* ATCC 35218 were also utilized as a control. Bacteria were routinely grown overnight in Luria-Bertani (LB) broth (0.5% yeast extract, 1% tryptone and 0.5% NaCl) incubated at 37°C in a shaker incubator (Thermo Scientific, Iowa City, IA, USA).

### Sample Collection

From January 2011 to December 2014 a total of 1,037 stool samples were collected from patients with diarrhea from 17 different municipalities of the Sinaloa State at Northwestern of Mexico. Inhabitants of these municipalities account for ∼99% of the State’s population (Supplementary Figure S1). Patients with acute diarrhea came to primary care units where they were evaluated by licensed physicians whom confirmed that patients had the passage of three or more loose or liquid stools per day or more frequent passage than is normal. Rectal swabs were collected by standard procedures for routine diagnostics, at the primary care units, and transferred to Cary-Blair transport medium and therefore no written consent was required from patients. Swabs were stored at 4°C at the communal health station until transportation in cold boxes to the laboratory for processing, usually within 2 h.

### Isolation and Identification of *E. coli* Strains

Stool swabs in Cary–Blair transport medium were directly inoculated onto MacConkey agar medium for the isolation of *E. coli*. Suspected *E. coli* colonies were further identified by biochemical tests API 20E^®^ (Biomeriux, USA). *E. coli* strains identified by biochemical tests were characterized by PCR. Strains were isolated from specimens obtained for routine testing of gastrointestinal pathogens at the mentioned hospitals thereby neither Institutional Review Board (IRB) approval was required nor inform consent was required from adult patients or parents or legal guardians of children.

### Preparation of Template DNA and Multiplex PCR to Identify DEC Strains

Five *E. coli* colonies per sample were analyzed. Strains were grown in 3 mL of LB broth for 18 h, pelleted by centrifugation at 10,000 *× g* for 10 min, and then resuspended in 0.3 ml of distilled water. Pellets were heated at 100°C for 10 min, vortexed for 10 s, and centrifuged again at 12,000 *× g* for 3 min. DNA-containing supernatants were transferred to 0.5 mL Eppendorf tubes and stored at -20°C until used. An aliquot of the DNA prep was used as a template in multiplex PCR reaction for the characterization of DEC strains as previously detailed by Canizalez-Roman et al. (2013).

### Adherence Assay on HEp-2 Cells

The adherence assay on HEp-2 cell was performed essentially as described by Cravioto et al. (1979). Briefly, HEp-2 cells grown in Eagle’s minimal essential medium (EMEM, GIBCO-BRL, Gaithers-burg, MD, USA), with 10% fetal calf serum and antibiotics were seeded on eight-well chamber slides (NUNC, Lab-Tek, USA) and incubated until reaching a confluence of 70–80% (∼2–3 days). The day of experiments, cells were washed twice with sterile PBS and then added with 0.5 ml of fresh EMEM medium without fetal bovine serum or antibiotics, but containing 0.5% (wt/vol) of D-mannose.

To prepare the inoculum, *E. coli* strains were grown overnight in 5 mL of LB broth, then centrifuged, washed twice with sterile PBS and resuspended in EMEM with no additives. One hundred microliters of the bacterial suspension (∼1.5 × 10^8^ CFU) were inoculated onto those HEp-2 cell cultures. Infected cells were incubated for 3 h at 37°C under a 5% CO_2_ atmosphere and then washed three times with sterile PBS, fixed with 70% methanol, and stained with Giemsa (Sigma-Aldrich, USA). Adherence patterns were assigned according to the description by Scaletsky et al. (1984, 1999) and Nataro et al. (1987). EPEC reference strain (E2349/68) showing localized adherence (LA), EAEC (042) that present aggregative adherence (AA), DAEC (Canizalez-Roman et al., 2013) that present diffusely adherence and *E. coli* K12 that does not attach to HEp-2 cells were included as a control.

### Antimicrobial Agent Susceptibility Testing

The Kirby-Bauer disk diffusion method was used to determine sensitivity or resistance to antimicrobial agents, according to guidelines developed by the Clinical Laboratory Standard Institute (CLSI) (CLSI, 2011). Recommendations by the National Antimicrobial Resistance Monitoring System for *E. coli* were utilized to define breakpoints of antibiotics and thus categorize the isolates as resistant, intermediate, or susceptible. These antibiotics were chosen on the basis of their use and availability to treat Gram negative infection in Mexico. The antibiotic sensi-disks (BD BBL, Sensi-Disc, Becton, Dickinson and Company, USA) used were the following: ampicillin (10 μg), tetracycline (30 μg), trimethoprim–sulfamethoxazole (SMTX 1.25 μg/23.75 μg), chloramphenicol (30 μg), nalidixic acid (30 μg), ciprofloxacin (5 μg), ceftazidime (30 μg), gentamicin (10 μg), and cefotaxime (30 μg). The protocol was performed as follows: fresh cultures were inoculated into LB broth and incubated until they reached a turbidity of 0.5 using the McFarland standard. Then, Mueller-Hinton agar plates were swabbed with these cultures and antibiotic disks (BD BBL, Franklin Lakes, NJ, USA) were placed onto inoculated plates under a sterile environment. The plates were incubated at 37°C for 18–20 h. Diameters (in millimeters) of clear zones of growth inhibition around the antimicrobial agent disks were measured using a precision digital caliper (Absolute, Mitutoyo, Japan). *E. coli* ATCC 25922 and *E. coli* ATCC 35218 obtained from the American Type Culture Collection (ATCC), and recommended by the CLSI, were used as a control.

### Statistical Analysis

Data were analyzed using Sigma Plot software (version 11.0; Systat Software, Inc., USA) and Epi Info^TM^ version 7. Percentages were compared using a Pearson chi-square test or Fisher’s exact test to establish mutual relatedness among the DEC pathogroups. *p*-values of 0.05 were considered as statistically significant and calculated the odds ratio (OR) and the 95% confidence interval (95% CI).

## Results

### Prevalence of Diarrheagenic *E. coli*

From the 1,037 stool samples collected from patients with acute diarrhea during the 4-year study period, DEC strains were isolated from 242 (23.3%) patients and identified using culture and the multiplex PCR protocol (**Table 1**). The most prevalent DEC pathotype isolated was EAEC accounting for 52% (*n* = 126) of all cases followed by EPEC [21.9% (*n* = 53)], ETEC [17.8% (*n* = 43)], DAEC [6.2% (*n* = 15)], STEC [1.23% (*n* = 3)], and EIEC [0.82% (*n* = 2)]. No EHEC strains were isolated from any of the examined stool samples collected (**Table 1**).

**Table 1:** Diarrheagenic *Escherichia coli* (DEC) isolated from diarrhea cases by age group.

Age group (years)	Diarrhea cases (*N* = 1,037)	Pathotype *n* (%)								Total
		EAEC *n* = 126 (52)		EPEC *n* = 53 (22)		ETEC *n* = 43 (18)	DAEC *n* = 15 (6)	STEC *n* = 3 (1)	EIEC *n* = 2 (0.8)	DEC (*n* = 242)
		Typical (*n* = 82)	Atypical (*n* = 44)	Typical (*n* = 17)	Atypical (*n* = 36)					
0–2	270 (26)	30 (11.1)^∗^	8 (3)	9 (3.3)	17 (6.3)	12 (4.4)	3 (1.1)	2 (0.7)	1 (0.4)	82 (30.3)^∗∗^
3–5	86 (8.3)	4 (4.7)	4 (4.7)	0	1 (1.2)	1 (1.2)	1 (1.2)	1 (1.2)	0	12 (14)
6–10	108 (10.4)	6 (5.6)	3 (2.8)	0	2 (1.9)	9 (8.3)	3 (2.8)	0	1 (0.9)	24 (22.2)
11–20	104 (10)	6 (5.8)	4 (3.8)	3 (2.9)	3 (2.9)	6 (5.8)	1 (1)	0	0	23 (22.1)
21–30	110 (10.6)	10 (9)	7 (6.4)	3 (2.7)	4 (3.6)	4 (3.6)	1 (0.9)	0	0	28 (25.5)
31–50	205 (19.8)	14 (6.8)	11 (5.4)	2 (1)	6 (2.9)	6 (2.9)	6 (2.9)	0	0	45 (22)
51–99	144 (13.9)	12 (8.3)	7 (4.9)	0	3 (2.1)	5 (3.5)	0	0	0	27 (18.8)
NP	10 (1)	1 (10)	0	0	0	0	0	0	0	1 (10)

*NP, not provided. ^∗^*p* = 0.048 in comparison with atypical EAEC. ^∗∗^*p* < 0.008 in comparison to all other age groups.*

Patients were categorized into seven age groups 0–2, 3–5, 6–10, 11–20, 21–30, 31–50, and 51–99 years and were evaluated for the prevalence of specific DEC. The age group from where more DEC strains were isolated from diarrhea cases was those children ≤2 years of age with 30.4% (*N* = 82) and of these almost half cases were produced by EAEC strains. DEC-induced diarrhea, in this age group, was statistically significant when compared to all others (**Table 1**). Prevalence of DEC in the others age groups ranged from 14 to 25.5% (**Table 1**).

EAEC, EPEC, and ETEC strains were isolated in all age groups (**Table 1**). EAEC strains were more frequently isolated from diarrhea cases of all age groups except for children age 6 through 10 years of age. In this age group ETEC strains were more frequently isolated (8.3%). Whereas typical EAEC strains were more prevalent than the atypical variant, atypical EPEC strains were more frequently isolated than typical variants from all age groups.

### Virulence Properties and Characterization of DEC Typical and Atypical Variants

EAEC strains were classified as typical if they carried a gene encoding for the transcriptional activator AggR (*agg*R), atypical EAEC strains do not encode this gene (Jenkins et al., 2006). The adhesion phenotype were assessed by the classic adherence assay on HEp-2 cells described by Scaletsky et al. (1984, 1999) and Nataro et al. (1987). To further characterize their virulence potential, we screened by PCR the presence of genes that have been implicated in virulence including the gene coding for dispersin (*aap*), aggregative adherence fimbria II (*aaf*II) and a marker for plasmid pCVD432 (Baudry et al., 1990; Nataro et al., 1993).

**FIGURE 1:**
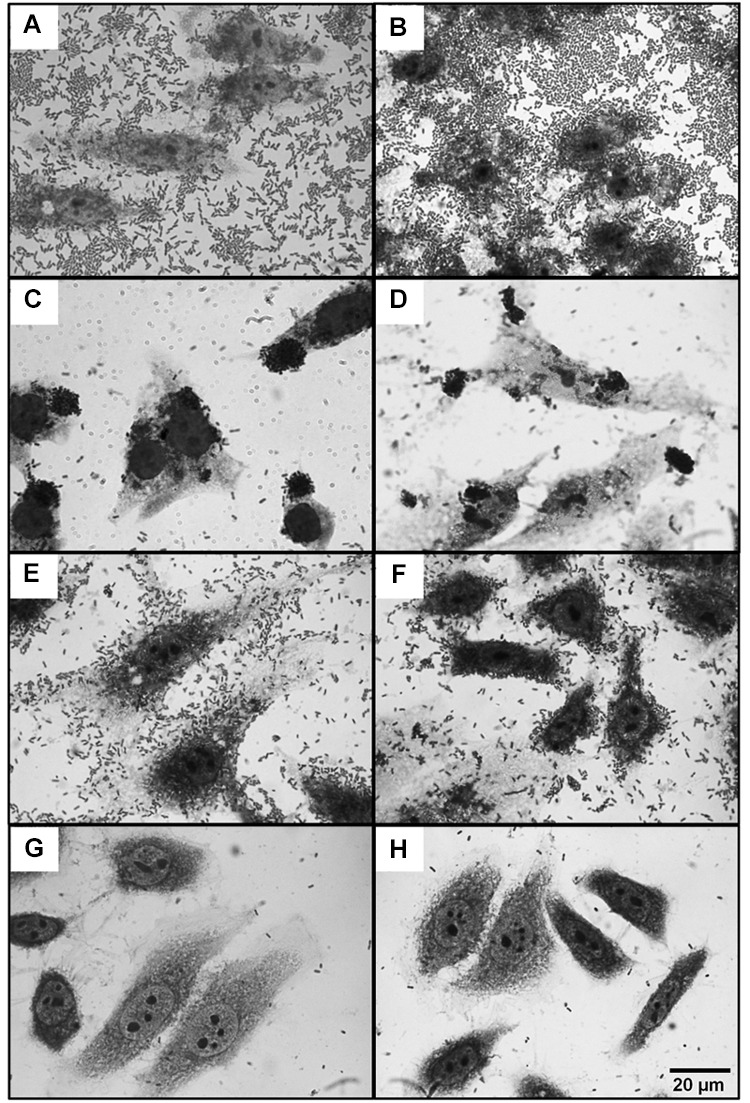
**Adherence phenotype of diarrheagenic *Escherichia coli* (DEC) on *in vitro*-cultured HEp-2 cells.** DEC cultures were used to infect HEp-2 cells for 3 h at 37°C, 5% CO_2_. Strains shown are **(A)** EAEC O42, **(B)** typical EAEC isolated from adult, **(C)** EPEC E2348/69 **(D)** typical EPEC isolated from children, **(E)** DAEC, **(F)** DAEC isolated from adolescent, **(G)** *E. coli* K12, and **(H)** STEC isolated from children. Cells were stained by Giemsa and photographed using a 40X objective.

Strains belonging to the typical EAEC (tEAEC) category were 82 (65.1%) whereas 44 (34.9%) strains were identified as atypical EAEC (aEAEC). In children ≤2 years, tEAEC strains were significantly more isolated than from any other age group (*p* < 0.008, for all comparisons). Moreover, tEAEC strains were significantly more isolated (*p* = 0.048) from children ≤2 years (11.1%) than aEAEC strains (3%). Only 48 typical EAEC strains (58.5%) and 16 aEAEC strains (36.3%) showed the characteristic AA pattern, i.e., stacked brick formation (**Figures 1A,B**; **Table 2**).

**Table 2:** Characterization of diarrheagenic *E. coli* based on the genotypic profile and adhesion phenotype.

Diarrheagenic *E. coli* group		Detection of virulence genes by PCR														Adhesion assays on HEp-2 cells				Total (*n* = 242)
		*eae*	*bfp*	*stx1*	*stx2*	*hlyA*	*aggR*	*aap*	*pCVD32*	*aafII*	*ipaH*	*virF*	*daaE*	*lt*	*stII*	LA	AggA	DA	NA	
EAEC (*n* = 126)	Typical (*n* = 82)						+	+	+	+						ND	5	ND	1	6
							+	+	+	-						ND	11	ND	11	22
							+	-	+	+						ND	3	ND	1	4
							+	-	+	-						ND	29	ND	21	50
	Atypical (*n* = 44)						-	+	+	+						ND	1	ND	1	2
							-	+	+	-						ND	10	ND	21	31
							-	-	+	+						ND	1	ND	0	1
							-	-	+	-						ND	4	ND	6	10
EPEC (*n* = 53)	Typical (*n* = 17)	+	+													14	ND	ND	3	17
	Atypical (*n* = 36)	+	-													5	ND	ND	31	36
ETEC														+	-	ND	ND	ND	43	43
DAEC													+			ND	ND	15	ND	15
STEC		-		+	-	-										ND	ND	ND	3	3
EIEC (*n* = 2)											+	-				ND	ND	ND	1	1
											+	+				ND	ND	ND	1	1

***-**/+: absence or presence of the gene. LA, localized adherence; AggA, aggregative adherence; DA, diffusely adherence; and NA, not adherent. ND, not detected.*

The prevalence of the different virulence genes detected in EAEC strains is shown in **Table 2**. We detected four different genotypes among tEAEC strains. The most prevalent was pCVD432+aggrR (*n* = 50; 61%) followed by pCVD432+aggr+*aap* (*n* = 22; 26.8%), pCVD432+aggrR+aap+aafII (*n* = 6; 7.3%) and pCVD432+aggrR+aafII (*n* = 4; 4.9%) genotype (**Table 2**). Likewise, aEAEC were grouped in four different genotypes, the most frequent was pCVD432+aap (*n* = 31; 70.5%) followed by pCVD432 (*n* = 10; 22.7%), pCVD432+aap+aafII (*n* = 2; 4.6%) and one strain encoding pCVD432+aafII (**Table 2**).

The second most prevalent DEC found in our study was EPEC (*n* = 53). Strains were classified as typical if they carried the intimin *eae* gene, and *bfp*A, whereas atypical strains encodes *eae* but not *bfp*A (Vidal et al., 2007). Our studies revealed that atypical EPEC strains (*n* = 36) were significantly more prevalent (*p* < 0.001) than typical strains (*n* = 17). While atypical EPEC strains were isolated from diarrhea cases in all age groups, children <2 years of age were more frequently affected by atypical strains (**Table 1**). As expected, most tEPEC strains [*n* = 14 (82.4%)] showed the LA pattern whereas only five aEPEC strains (13.9%) showed the LA phenotype (**Figures 1C,D**; **Table 2**).

On the other hand, all DAEC strains showed the characteristic diffuse adherence pattern (**Figures 1E,F**; **Table 2**) whereas STEC, EIEC, and ETEC isolated in this study from patients with diarrhea did not adhere to HEp-2 cells within the 3 h incubation period (**Figures 1G,H**; **Table 2**).

### Distribution of DEC Strains in the Different Surveyed Regions of the Sinaloa State

**FIGURE 2:**
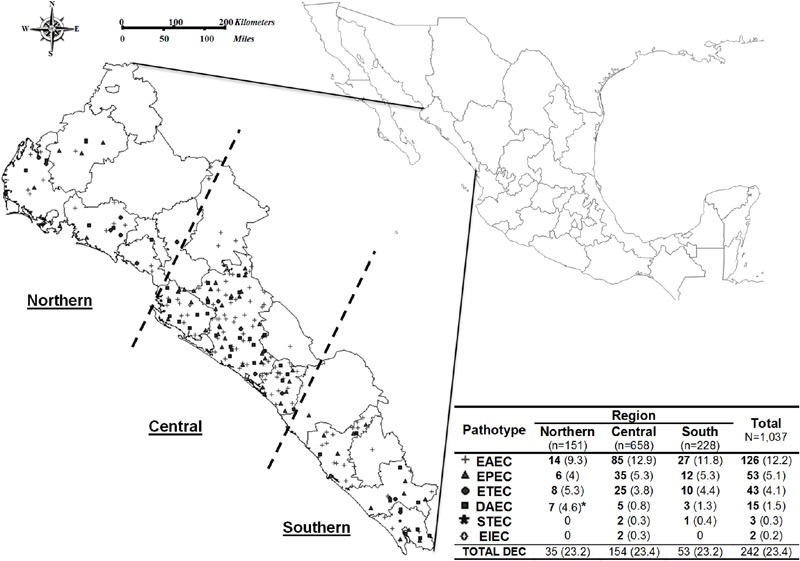
**Diarrheagenic *Escherichia coli* distribution was described in three geographical zone.** Northern (Incluide the municipalities of Ahome, El Fuerte, Choix, Sinaloa de Leyva, Guasave, Mocorito and Angostura), Central (Salvador Alvarado, Culiacan, Navolato, Badiraguato, Cosala and Elota) and South (San Ignacio, Mazatlan, El Rosario, Concordia and Escuinapa).

To determine the distribution of DEC pathotypes among the three different regions in Sinaloa, we compared the number of DEC pathotypes and commensal *E. coli* in the northern, central and south region (**Figure 2**). Statistical analysis revealed no differences (*p* > 0.950; **Figure 2**) in the prevalence of DEC types when the different regions were compared each other, the prevalence was similar ∼24% across regions. Accordingly, the prevalence of EAEC, EPEC, and ETEC strains isolated in the northern, central and south was not significantly different (*p* = 0.218). However, the prevalence of DAEC strains isolated in the northern (4.6%) was statistically significant to that in the central region (0.8%; *p* < 0.05) but not to that in the south (1.3%; *p* = 0.0996). STEC strains was only isolated from cases at the central and south regions (0.3 and 0.4%, respectively) and EIEC strains were only isolated from cases detected at the central region (0.3%; **Figure 2**).

### Antibiotic Susceptibility of DEC Strains Isolated from Diarrhea Cases

Antibiotic resistance of DEC strains to nine antibiotics was evaluated and is shown in **Table 3**. These studies demonstrate that >65% of DEC strains were resistant to tetracycline, ampicillin, and SMTX. A lower prevalence (<35%) of resistance was demonstrated for nalidixic acid (33.1%), cefotaxime (28.9%), chloramphenicol (23.6%), ciprofloxacin (21.1%), gentamicin (19.8%), and ceftazidime (7.9%).

**Table 3:** Antimicrobial resistance among DEC strains isolated from diarrhea cases.

Class/antibiotic	Total (*n* = 242)	DEC category *n* (%) resistance strains					
		EAEC (*n* = 126)	EPEC (*n* = 53)	ETEC (*n* = 43)	DAEC (*n* = 15)	STEC (*n* = 3)	EIEC (*n* = 2)
**Aminoglycoside**							
*Gentamicin*	48 (19.8)	20 (15.9)	7 (13.2)	12 (27.9)ˆ*	**7 (46.7)^∗^**	1 (33.3)	1 (50)
**Quinolones and Fluoroquinolones**							
*Ciprofloxacin*	51 (21.1)	19 (15.1)	**24 (45.3)^∗^**	5 (11.6)	3 (20)	0	0
*Nalidixic acid*	80 (33.1)	38 (30.2)	**29 (54.7)^∗^**	8 (18.6)	5 (33.3)^∗^	0	0
**Sulfonamides and potentiated sulfonamides**							
*Sulfamethoxazole-trimethoprim*	158 (65.3)	86 (68.3)	**44 (83)^∗^**	16 (37.2)	11 (73.3)^∗^	0	1 (50)
**Tetracyclines**							
*Tetracycline*	176 (72.7)	92 (73)	**50 (94.3)^∗^**	19 (44.2)	11 (73.3)	2 (75)	2 (100)
**Beta lactams**							
*Ampicillin*	170 (70.3)	86 (68.3)	**44 (83)^∗^**	26 (60.5)	12 (80)^∗^	2 (75)	0
**Cephalosporins**							
*Ceftazidime*	19 (7.9)	4 (5)	6 (11.3)	3 (7)	**5 (33.3)^∗^**	0	0
*Cefotaxime*	70 (28.9)	26 (20.6)	17 (32.1)	16 (37.2)	**8 (53.3)^∗^**	1 (33.3)	2 (100)
**Phenicols**							
*Chloramphenicol*	57 (23.6)	23 (18.3)	**26 (49.1)^∗^**	5 (11.6)	3 (20)^∗^	0	0

*Bold indicates the category with major proportion of resistant strains; ^∗^*p* < 0.05 statistical significance of resistance compared with each other pathotype.*

When specific DEC categories were compared, we observed that EPEC strains were significantly more resistant (*p* < 0.05) to tetracycline (94.3% of resistance), than DAEC, EAEC, and ETEC (73.3, 73, and 44.2% of resistance respectively). EPEC and DAEC strains presented the highest rates of resistance to ampicillin, SMTX, nalidixic acid, chloramphenicol, and ciprofloxacin (**Table 3**). Rates of resistance among DAEC strains were significantly higher than that of EAEC, EPEC, and ETEC to cephalosporins (cefotaxime and ceftazidime) and gentamicin (*p* < 0.05, **Table 3**). Of the few STEC strains isolated, two strains were resistant to ampicillin and tetracycline but only one to cefotaxime and gentamicin. EIEC strains (*n* = 2) were resistant to tetracycline and cefotaxime whereas all STEC and EIEC strains were susceptible to ciprofloxacin, ceftazidime, nalidixic acid, and chloramphenicol (**Table 3**).

### Multidrug Resistance of DEC Strains Isolated from Patients with Acute Diarrhea

Multiple comparisons showed a significant difference in DEC strains susceptible, resistant, and multidrug resistant (MDR; resistance to two or more antibiotics tested). From all DEC strains isolated 9.1% (*n* = 22) were susceptible to all antibiotics evaluated, ∼91% (*n* = 220) strains were resistance at least one antimicrobial and ∼81% (*n* = 194) were MDR (**Table 4**). Among DEC isolates with a MDR phenotype our studies found different phenotypes, however, the more prevalent was the DEC resistant to three antimicrobial agents (52; 21.5%), followed by strains that were resistant to four antibiotics (48; 19.8%), to six (32; 13.2%), to five (28; 11.6%), to two (24; 9.9%) and the less frequent phenotype was resistance to seven antimicrobial agents (7; 2.9%), eight antibiotics (2; 0.8%) and only one EAEC strain was resistant to all nine antimicrobial agents evaluated (**Table 4**).

**Table 4:** Multiple antimicrobial resistance of DEC strains isolated from diarrhea cases.

Category	Resistant to *n* antibiotics	Total 242	DEC strains *n* (%)					
			EAEC 126 (52)	EPEC (*n* = 53)	ETEC (*n* = 43)	DAEC (*n* = 15)	STEC (*n* = 3)	EIEC (*n* = 2)
Susceptible	0	22 (9.1)	14 (11.1)	1 (1.9)	6 (13.9)	0	1 (33.3)	0
Resistance	1	26 (10.7)	15 (11.9)	2 (3.8)	8 (18.6)	1 (6.7)	0	0
MDR	2	24 (9.9)	11 (8.7)	3 (5.7)	8 (18.6)	1 (6.7)	0	1 (50)
	3	52 (21.5)	31 (24.6)	9 (16.9)	7 (16.3)	3 (20)	2 (66.6)	0
	4	48 (19.8)	28 (22.2)	10 (18.9)	7 (16.3)	2 (13.3)	0	1 (50)
	5	28 (11.6)	15 (11.9)	3 (5.7)	5 (11.6)	5 (33.3)	0	0
	6	32 (13.2)	9 (7.1)	20 (37.7)	2 (4.7)	1 (6.7)	0	0
	7	7 (2.9)	1 (0.8)	4 (7.5)	0	2 (13.3)	0	0
	8	2 (0.8)	1 (0.8)	1 (1.9)	0	0	0	0
	9	1 (0.4)	1 (0.8)	0	0	0	0	0

*MDR phenotype demonstrate that EPEC or DAEC strains were significantly (*p* < 0.05) higher than EAEC and ETEC strains.*

The prevalence of the specific DEC pathotype with MDR phenotype demonstrate that EPEC (94%) or DAEC (93%) strains were significantly higher than EAEC, and ETEC strains which had a prevalence of 77 and 67.4%, respectively (*p* < 0.05; **Table 4**). While STEC and EIEC strains showed a high prevalence of MDR, 66.6 and 100%, respectively, statistical analysis was not performed due to the small sample size.

A detailed analysis of co-resistance to antibiotics was made and is shown in **Table 5**. The more prevalent co-resistance observed for DEC strains was tetracycline+ampicillin (*n* = 143, 81.3%), followed by tetracycline+SMTX (*n* = 141, 80.1%), ampicillin+SMTX (*n* = 138, 81.1%), chloramphenicol+SMTX (*n* = 47, 82.5%) and, as expected given they are quinolones, ciprofloxacin+nalidixic acid (*n* = 41, 80.4%). Each of these combinations were significantly more prevalent than all others (*p* < 0.05). More prevalent co-resistance combinations to three antibiotics was tetracycline+ampicillin+SMTX (*n* = 125, 87.4%), co-resistance to four antibiotics tetracycline+ampicillin+SMTX+chloramphenicol (*n* = 42, 82.4%) and to five antibiotics was tetracycline+ampicillin+chloramphenicol+gentamicin+SMTX (*n* = 8, 88.9%) (*p* < 0.05).

**Table 5:** Co-resistance to antibiotics of DEC strains isolated from diarrhea cases in Sinaloa during 2011–2014.

	Total	AMP	Cm	GEN	SMTX	CEF	CAZ	CIP	NA
(A) TET	176	143 (81.3)^∗^	55 (31.3)	39 (22.2)	141 (80.1)ˆ*	53 (30.1)	13 (7.4)	48 (27.3)	69 (39.2)
(B) AMP	170		53 (31.2)	37 (21.8)	138 (81.1)ˆ*	52 (30.5)	17 (10)	44 (25.9)	68 (40)
(C) Cm	57			10 (17.5)	47 (82.5)ˆ*	18 (31.6)	7 (12.3)	22 (38.6)	35 (61.4)
(D) GEN	48				32 (66.7)	24 (50)	6 (12.5)	15 (31.3)	21(43.8)
(E) SMTX	158					43 (27.2)	12 (7.59)	45 (28.5)	67 (42.4)
(F) CTX	70						14 (20)	11 (15.7)	22 (31.4)
(G) CAZ	19							2 (10.5)	7 (36.8)
(H) CIP	51								41 (80.4)
(I) NA	80								

AB	143		51 (35.7)	34 (23.8)	125 (87.4)ˆ*	44 (30.8)	12 (8.4)	42 (29.4)	61 (42.7)
ABC	51			9 (17.7)	42 (82.4)ˆ*	18 (35.3)	7 (13.7)	20 (39.2)	32 (62.7)
ABCD	9				8 (88.9)ˆ*	6 (66.7)ˆ*	2 (22.2)	3 (33.3)	4 (44.4)
ABCDE	8					5 (62.5)	2 (25)	3 (37.5)	4 (50)
ABCDEF	5						1 (20)	3 (60)	3 (60)
ABCDEFG	0							0	0
ABCDEFGH	1								1 (100)

*^∗^Statistical significance (*p* < 0.05) co-resistance combinations. TET, tetracycline; AMP, ampicillin; Cm, chloramphenicol; GEN, gentamicin; SMTX, sulfamethoxazole-trimethoprim; CTX, cefotaxime; CAZ, ceftazidime; CIP, ciprofloxacin; NA, nalidixic acid.*

## Discussion

Diarrheagenic *E. coli* is a potential public health risk for adults and children in developing countries, causing diarrhea. In this study we evaluated the frequency of DEC categories, virulence markers and antibiotic resistance patterns in patients with acute diarrhea in Sinaloa, Mexico. Before this study little to nothing was known about the presence of DEC strains in diarrheal diseases of children, adults and the elderly in the northwest of Mexico. We isolated the different pathogroups of DEC from all age groups.

The frequency of DEC categories found in our study (23%) was similar to other reports from developing countries (Bueris et al., 2007; Moyo et al., 2007; Alikhani et al., 2013); EAEC was the most commonly isolated category, followed by EPEC and ETEC. These three pathotypes of diarrheagenic *E. coli* were detected most frequently in children with acute diarrhea in Brazil (Bueris et al., 2007), Lybia (Ali et al., 2012), and Tanzania (Moyo et al., 2007). In addition, we identified DAEC, STEC and observed a very low frequency of EIEC. Previous studies from Kenya and Brazil reported similar findings in children (diarrhea) with prevalence of less than 1% for STEC and EIEC (Franzolin et al., 2005; Makobe et al., 2012). All STEC strains identified in this study were negative for the O157:H7 serotype. Similar reports indicate the Non-O157:H7 STEC strains have been described in Mexico (Estrada-Garcia et al., 2009) and other Latin American countries such as Brazil (Bueris et al., 2007) and Colombia (Rugeles et al., 2010).

EAEC is an emerging pathogen responsible for acute and persistent diarrhea, this pathotype may cause malnutrition and growth defects in children. EAEC strains have been associated with traveler’s diarrhea in both developing and industrialized countries (Kaper et al., 2004; Kaur et al., 2010). The high frequency of EAEC described in our study, along with the high resistance to antibiotics, support the need for follow-up epidemiological studies, pathogenesis, and its role in the different forms of diarrhea. On the other hand, EPEC was the second most frequently isolated pathotype. Generally, EPEC are among the most important pathogens infecting children under 2 years of age in the developing world (Trabulsi et al., 2002; Nguyen et al., 2006; Nakhjavani et al., 2013).

The incidence of different pathogroups of DEC after this surveillance study, was not uniform in all age groups. In this sense we found a high presence of EAEC cases in children less than 2 years and young adults ≥21 years of age. In previous studies of acute diarrhea of U.S. students (≥21 years of age) traveling to Mexico, researchers found that the prevalence of EAEC was higher than that of the other pathotypes (Paredes-Paredes et al., 2011). On the other hand, EPEC and STEC were detected more frequently in children less than 5 years of age but ETEC and EIEC were isolated from children of 6–10 years of age. Several studies with stool samples from children under five showed similar results to the ones demonstrated in our study (Franzolin et al., 2005; Amisano et al., 2011; Makobe et al., 2012). Whereas, the frequency of DAEC strains in our study was in general low, DAEC strains have been recovered frequently from adults with diarrhea in Brazil (Mansan-Almeida et al., 2013). Furthermore, in Brazilian children aged 2–5 years living in low socioeconomic level, DAEC strains were the second most frequently isolated pathotype associated with diarrhea (Lozer et al., 2013).

The majority of tEAEC found in the study showed an AA pattern and a low percentage of aEAEC also showed a typical AA pattern. This data suggests some variations in the adherence ability of EAEC and the presence of unknown adhesive fimbriae. For example, mutations within a fimbrial gene (*fimH)*, of a new *E. coli* pathotype referred to as adherent-invasive *E. coli* (AIEC), have been demonstrated to enhance adhesion to intestinal cells (Iebba et al., 2012). Further investigations are needed to detect other virulence genes which are specific to atypical EAEC strains found in patients with acute diarrhea at northwest of Mexico.

Atypical EPEC is an emergent enteric pathogen that has only recently begun to attract the attention of investigators (Afset et al., 2004; Vidal et al., 2007; Nakhjavani et al., 2013). In our study we found that aEPEC were more prevalent than tEPEC with LA phenotype in HEp-2 cells, similar results were found in children less than 2 years old in Mexico (Estrada-Garcia et al., 2009), and epidemiological studies in several countries showed that the atypical EPEC strains have become a more frequent cause of diarrhea than typical EPEC (Trabulsi et al., 2002; Afset et al., 2004; Franzolin et al., 2005; Vidal et al., 2005). These observations have been linked to the duration of the diarrheal disease (Afset et al., 2004; Nguyen et al., 2006).

The observed resistance to antibiotics in our study may be a result of misuse of antibiotics for treating infectious diseases. Supporting this hypothesis, most strains were resistant to the most accessible, and known by the general population, antibiotics such as ampicillin, tetracycline, and TMSX. These drugs are highly prescribed in some countries as a treatment for enteric infections caused by Gram-negative bacteria (Livermore et al., 2002; Yang et al., 2009). Furthermore, Sinaloa has an important food industry and agricultural resources that may utilize excessive antibiotics stimulating this way the development of resistance (Hawkey and Jones, 2009). This trend has been observed in other developing countries, such as Iran, where DEC strains isolated from adults were resistant for ampicillin, co-trimoxazole, and tetracycline with 67% of DEC strains being multidrug-resistant (Alikhani et al., 2013). Accordingly, strains isolated from Iranian children with diarrhea were resistant to ampicillin and tetracycline (89 and 83%, respectively) with all of DEC isolated from this particular population presenting the MDR phenotype (Heidary et al., 2014). In Mexico, Estrada-Garcia et al. (2005) found in a study carried out in children that 70% of DEC strains were resistant to ampicillin and SMTX; whereas most strains were sensitive to ciprofloxacin and cefotaxime.

The two most prevalent multi-drug resistance patterns among the DEC isolates demonstrated in this study were tetracycline and ampicillin followed by tetracycline, ampicillin and trimethoprim–sulfamethoxazole. Ochoa et al. (2009) reported multi-resistance to ampicillin and trimethoprim–sulfamethoxazole as the second most common pattern in DEC strains isolated in Peru, and EI Metwally (Ei Metwally et al., 2007) reported that 56% of DEC isolates were multi-drug resistant simultaneous against, trimethoprim–sulfamethoxazole, ampicillin and tetracycline.

In a previous research article, we reported the prevalence of DEC strains from several food items for human consumption in the state of Sinaloa. We demonstrated that EPEC and EAEC were the most prevalent pathotypes with ∼70% of strains showing resistance to antibiotics (Canizalez-Roman et al., 2013). Accordingly, these two pathotype were the most isolated in the curretn survey from patients with acute diarrhea. As per those strains isolated from food items, straisn from patient with diarrhea showed a high resistance to first-line antibiotics. DEC strains have been associated with foodborne illnesses (Nataro and Kaper, 1998) and this bacterial DEC burden in Sinaloa’s food samples may be related to cause human disease found in this study. Molecular studies are currently underway in our laboratories to investigate genetic associations between strains isolated from food items and those isolated in this study from patients with diarrhea.

To the best of our knowledge this is the first study to demonstrate that DEC strains, particularly EAEC and EPEC, might contribute to the burden of diarrheal diseases in children, adults and the elderly in the northwest of Mexico. A comprehensive study of prevalence with molecular methodology is warranted to completely define the role of DEC strains in cases of acute diarrhea. We, however, advice to include improved detection methods for bacterial diarrheal pathogens, such as DEC strains, and appropriate studies of antimicrobial resistance in order to best manage cases of acute diarrhea in this region and beyond and, equally important, to assistant alleviating the increasing prevalence of MDR of DEC strains.

## Author Contributions

AC-R and NL-S conceived and designed the study. EG-N, HF-V, AC-R, and JV-R collected the samples and performed the laboratory analyses. JV, SM-A, GA-C, JD-Q, and NL-S analyzed the data and HF-V, AC-R, and JV drafted the first version of the manuscript. All authors revised the manuscript critically and contributed to the final version.

## Conflict of Interest Statement

The authors declare that the research was conducted in the absence of any commercial or financial relationships that could be construed as a potential conflict of interest.
