# Supplementary material for: Surveillance of Diarrheagenic Escherichia coli Strains Isolated from Diarrhea Cases from Children, Adults and Elderly at Northwest of Mexico
Source: Front Microbiol. 2016 Nov 30;7:1924. doi: 10.3389/fmicb.2016.01924 (PMC5127799; doi:10.3389/fmicb.2016.01924)
Supplement: Supplementary file 1 [file Image_1.PDF]

Supplementary Material

Surveillance of Diarrheagenic Escherichia coli strains isolated from diarrhea cases at northwest of Mexico

Adrian Canizalez-Roman*, Héctor Flores-Villaseñor, Edgar Gonzalez-Nuñez, Jorge Velazquez-Roman, Jorge E. Vidal, Secundino Muro-Amador, Gerardo Alapizco-Castro, Alberto Díaz-Quiñonez and Nidia León-Sicairos.

***Correspondence:** Corresponding Author: canizalez@uas.edu.mx

Supplementary Figures

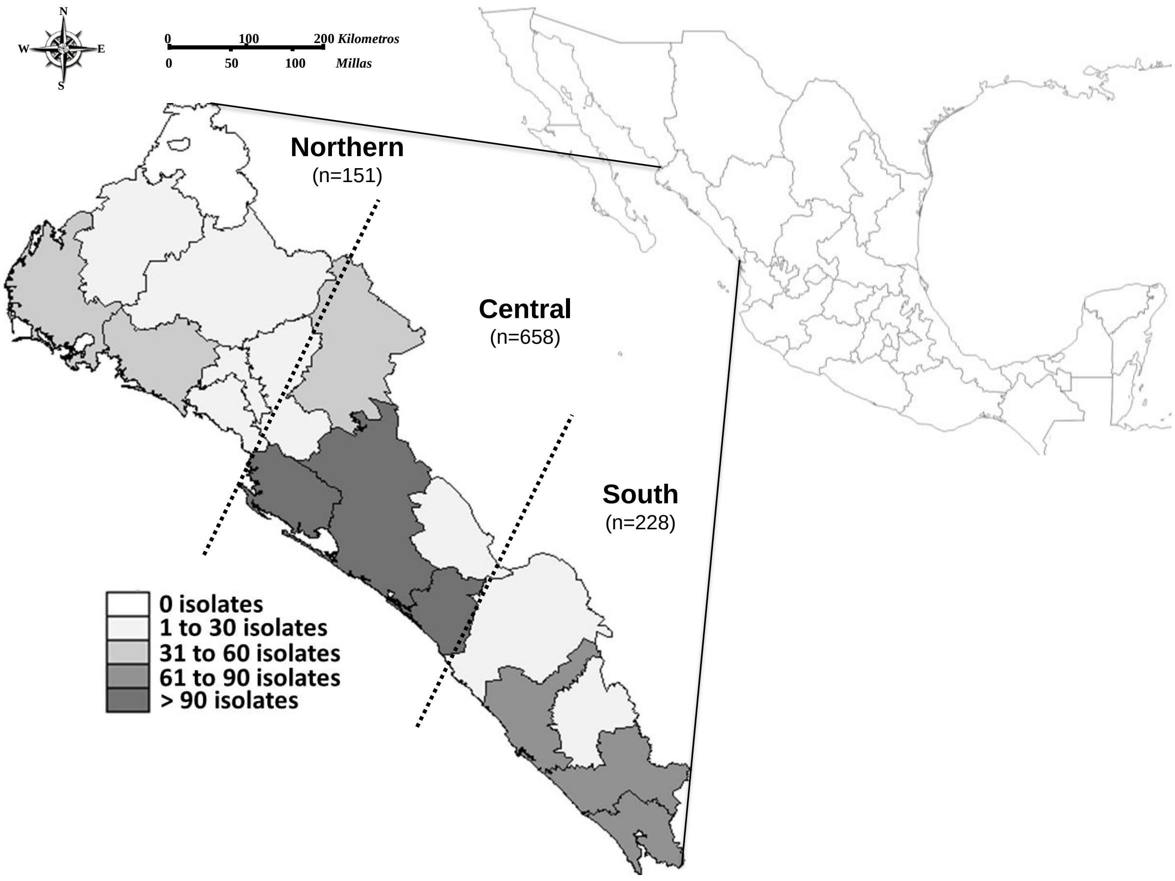

Supplementary Figure 1. Diagram of the total stool samples collected from January 2011 to December 2014 in seventeen different municipalities of the Sinaloa state at Northwest of Mexico. The location was described in three geographical zone. Northern (Include the municipalities of Ahome, El Fuerte, Choix, Sinaloa de Leyva, Guasave, Mocorito and Angostura), Central (Salvador Alvarado, Culiacan, Navolato, Badiraguato, Cosala and Elota) and Southern (San Ignacio, Mazatlan, El Rosario, Concordia and Escuinapa).
